# Comparing the efficacy of induction-concurrent with concurrent-adjuvant chemotherapy in locoregionally advanced nasopharyngeal carcinoma: a propensity score matching analysis

**DOI:** 10.18632/oncotarget.20389

**Published:** 2017-08-22

**Authors:** Li-Rong Wu, Xue-Song Jiang, Xue Song, Hong-Liang Yu, Yan-Xin Fan, Fei-Jiang Wang, Sheng-Fu Huang, Wen-Jie Guo, Xia He, Ju-Ying Liu

**Affiliations:** ^1^ Department of Radiation Oncology, Nanjing Medical University Affiliated Cancer Hospital, Jiangsu Cancer Hospital and Jiangsu Institute of Cancer Research, Nanjing 210009, China

**Keywords:** nasopharyngeal carcinoma, locoregionally advanced, induction chemotherapy, adjuvant chemotherapy, intensity-modulated radiotherapy

## Abstract

**Purpose:**

This study aimed to compare the efficacy of induction-concurrent (IC-CCRT) with concurrent-adjuvant (CCRT-AC) chemotherapy in patients with locoregionally advanced nasopharyngeal carcinoma (LA-NPC) treated by intensity-modulated radiotherapy (IMRT).

**Materials and Methods:**

Data on 834 patients with newly diagnosed, non-metastatic stage III-IVA (except T3N0) NPC receiving either IC-CCRT or CCRT-AC between July, 2004 and December, 2014 were retrospectively reviewed. Propensity score matching (PSM) method was adopted to balance prognostic factors and match patients. Survival outcomes of matched patients between IC-CCRT and CCRT-AC were compared.

**Results:**

The median follow-up duration is 45.2 months (range, 1.07–145.4 months). Overall, 309 pairs were selected by PSM. Univariate analysis revealed the CCRT-AC group achieved significantly higher 3-year DFS (83.9% vs. 78.7 %; *P* = 0.014) and OS (87.6% vs. 87.0%; *P* = 0.031). Multivariate analysis also identified treatment group (IC-CCRT vs. CCRT-AC) as an independent prognostic factor for 3-year DFS (HR, 1.546; 95% CI, 1.113–2.149; *P* = 0.009) and OS (HR, 1.487; 95% CI, 1.035–2.136; *P* = 0.032). Subgroup analysis revealed IC-CCRT was a protective factor for DMFS (HR, 0.145; 95% CI, 0.043–0.488; P = 0.002) in stage III disease; however, it could adversely affected DFS (HR, 2.009; 95% CI, 1.316–3.065; *P* = 0.001), OS (HR, 1.671; 95% CI, 1.060–2.636; *P* = 0.027) and DMFS (HR, 1.986; 95% CI, 1.155–3.416; *P* = 0.013) in stage IVA disease.

**Conclusions:**

CCRT-AC may be a more effective treatment modality in patients with stage IVA NPC disease, while IC-CCRT was superior in stage III disease.

## INTRODUCTION

As an Epstein-Barr virus (EBV)-associated malignant tumor [1–3], nasopharyngeal carcinoma (NPC) has an extremely unbalanced distribution: 86700 new cases were reported with 71% of those occurring in east and southeast parts of Asia in 2012 [4]. Apart from other head and neck cancers, radiotherapy is the only curative treatment modality for non-metastatic NPC due to its radiosensitivity and anatomical constraint which makes radical surgery unavailable. Also, NPC is highly sensitive to chemotherapy, and combined chemotherapy-radiotherapy strategies have been documented to achieve better survival outcomes than radiotherapy alone in advanced NPC [5–12]. Consequently, concurrent chemoradiotherapy (CCRT) has become the basic treatment for locoregionally advanced NPC (LA-NPC). However, CCRT may be insufficient as survival of LA-NPC is mainly compromised by distant metastasis, especially for patients receiving intensity-modulated radiotherapy (IMRT) [13, 14]. Therefore, additional chemotherapy to CCRT is urgently needed.

Since the Intergroup-0099 trial [5] showed a benefit of 3-year overall survival provided by CCRT plus adjuvant chemotherapy (AC), this regimen has been recommended as the standard care for advanced NPC. However, most patients suffering severe toxicities during CCRT and could not tolerate the toxicities of AC, which constrains its wide use. Therefore, a strategy with better efficacy and less toxicities should be developed to improve compliance to treatment and systemic control. Induction chemotherapy (IC), delivered before CCRT, has attracted a lot of attention as it has better compliance and could eliminate micro-metastasis. For the last twenty years, much efforts have been made regarding the value of IC [15–23]; however, most of these studies achieved negative results except for the studies by Hui et al. [19] and by Sun et al. [23]. Besides, a pooled data analysis by Chua et al. [24] revealed IC was associated with significantly decreased disease-specific survival, which indicating that IC may still play an important role in LA-NPC.

Given the abovementioned evidence, there has come a concern: which chemotherapy sequence is better? Induction with concurrent (IC-CCRT) or concurrent followed by adjuvant (CCRT-AC)? However, little is known about the head-to-he comparison of these two regimens except the results from two network meta-analysis [25, 26]. Accordingly, we conducted this retrospective study to compare the survival difference between these treatment modalities.

## RESULTS

### Baseline characteristics

From July 2004 to December 2014, we identified 834 patients with LA-NPC (except T3N0) receiving either IC-CCRT or CCRT-AC, among whom 487 (58.4%) received IC-CCRT and 347 (41.6%) received CCRT-AC. For the whole cohort, the male (*n* = 641)-to-female (*n* = 193) ratio was 3.3:1, and the median age was 48 (18–70) years-old. After matching by PSM, 309 pairs were selected from the original 834 patients and the baseline characteristics were summarized in Table 1. Obviously, patients in these two groups received similar treatment intensity (chemotherapy regimen and cycle). Moreover, host and tumor stage factors were also well balanced between these two groups after matching (*P* > 0.05 for all rates).

**Table 1:** Baseline characteristics of the 309 pairs with LA-NPC (except T3N0)

Characteristics	IC + CCRT No. (%)	CCRT + AC No. (%)	*P*
Median age (y, range)	47 (18–70)	48 (18–69)	0.213^a^
Gender			0.296^b^
Male	258 (83.5)	248 (80.3)	
Female	51 (16.5)	61 (19.7)	
KPS			0.468^b^
≥ 90	222 (71.8)	230 (74.4)	
≤ 80	87 (28.2)	79 (25.6)	
Smoking			0.934^b^
Yes	116 (37.5)	117 (37.9)	
No	193 (62.5)	192 (62.1)	
Drinking			0.378^b^
Yes	53 (17.2)	45 (14.6)	
No	256 (82.8)	264 (85.4)	
T category^c^			0.348^b^
T1	19 (6.1)	30 (9.7)	
T2	25 (8.1)	25 (8.1)	
T3	130 (42.1)	116 (37.5)	
T4	135 (43.7)	138 (44.7)	
N category^c^			0.470^b^
N0	18 (5.8)	15 (4.9)	
N1	164 (53.1)	153 (49.5)	
N2	89 (28.8)	107 (34.6)	
N3	38 (12.3)	34 (11.0)	
Overall stage^c^			0.835^b^
III	142 (46.0)	146 (47.2)	
IVA	167 (54.0)	163 (52.8)	
CCRT regimen			0.489^b^
PF	94 (30.4)	102 (33.0)	
TP	215 (69.6)	207 (67.0)	
IC/AC cycles			0.747^b^
2	170 (55.0)	166 (53.7)	
3	139 (45.0)	143 (46.3)	
IC/AC regimen			0.723^b^
TPF	88 (28.5)	94 (30.4)	
TP	156 (50.5)	146 (47.2)	
PF	65 (21.0)	69 (23.4)	

Abbreviations: LA-NPC = locoregionally advanced nasopharyngeal carcinoma; IC = induction chemotherapy; CCRT = concurrent chemoradiotherapy; AC = adjuvant chemotherapy; KPS = karnofsky performance score; IMRT = intensity-modulated radiotherapy.

^a^*P*-value was calculated by Non-parametric test.

^b^*P*-values were calculated by Chi-square test.

^c^According to the 8th AJCC/UICC staging system.

### Treatment failure patterns

Up to the last visit (July 2016), the median follow-up duration was 45.2 months (range, 1.07–145.4 months) for the selected cohort. Notably, 73 (23.6%) patients in the IC-CCRT group and 49 (15.9%) in the CCRT-AC group died (*P* = 0.015). Among the 122 deaths, there were 16 (13.1%) non-cancer deaths with 12 (9.8%) and 4 (3.3%) in IC-CCRT and CCRT-AC groups, respectively. Moreover, 48 (15.5%) patients in IC-CCRT group and 39 (12.6%) in the CCRT-AC group developed distant metastasis (*P* = 0.298). Particularly, 30 (9.7%) in IC-CCRT group and 20 (6.5%) in CCRT-AC group experienced locoregional recurrence (*P* = 0.140). Overall, 87 (28.2%) patients in the IC-CCRT and 60 (19.4%) in CCRT-AC group suffered treatment failure (*P* = 0.011).

### Univariate and multivariate analysis

**Figure 1:**
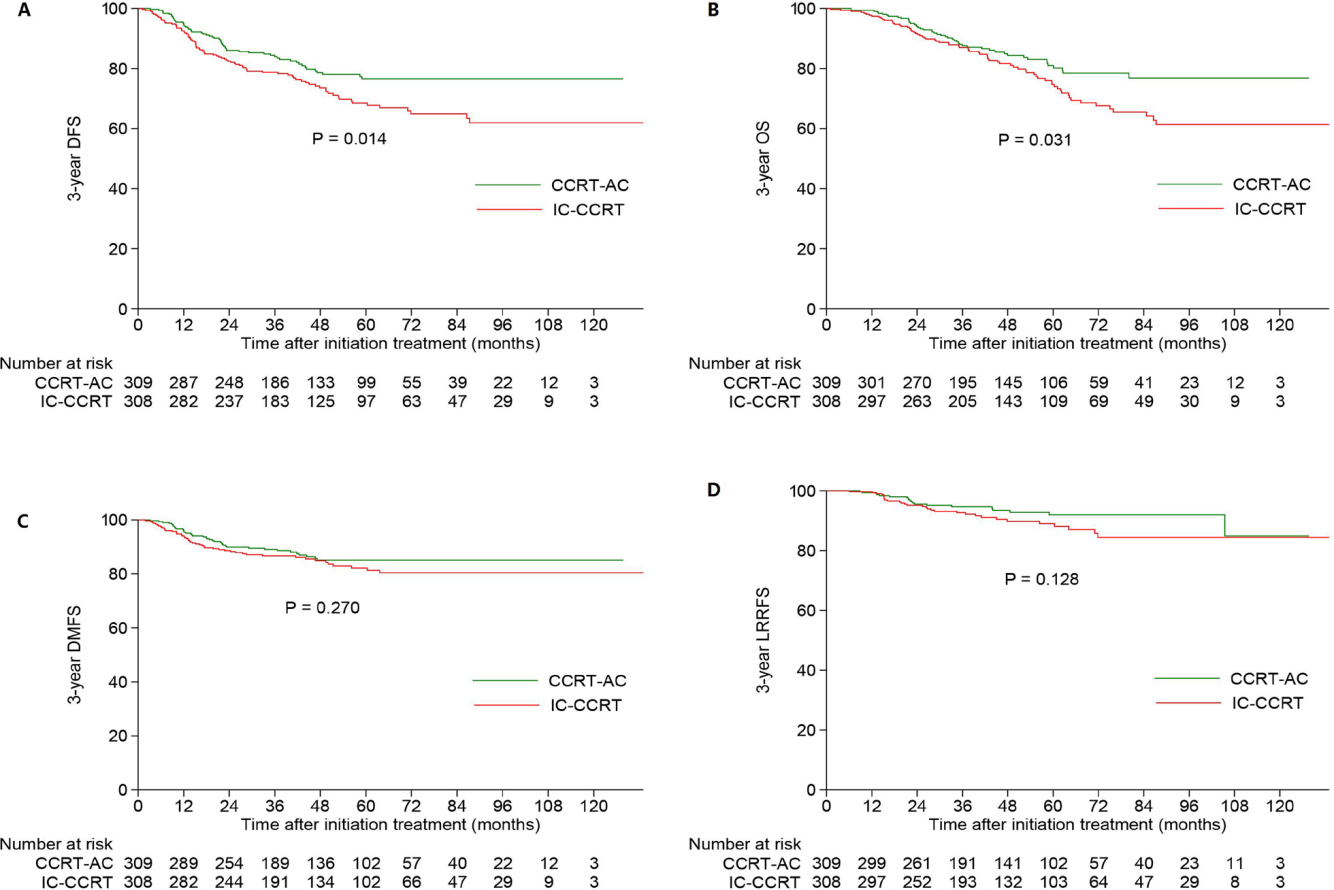
Kaplan-Meier DFS (**A**) OS (**B**), DMFS (**C**), and LRRFS (**D**) curves for 309 pairs of patients stratified as IC-CCRT and CCRT-AC groups. Abbreviations: OS = overall survival; DFS = disease-free survival; DMFS = distant metastasis-free survival; LRRFS = locoregional relapse-free survival; IC = induction chemotherapy; CCRT = concurrent chemoradiotherapy; AC = adjuvant chemotherapy.

Univariate analysis revealed the estimated 3-year DFS, OS, DMFS and LRRFS rates were 81.3%, 87.3%, 87.9% and 93.7% for the whole cohort. Compared with the CCRT-AC group, the IC-CCRT group achieved significantly worse 3-year DFS (78.7% vs. 83.9%, *P* = 0.014; Figure 1A) and OS (87.0% vs. 87.6%, *P* = 0.031; Figure 1B); however, 3-year DMFS (86.7% vs. 89.1%, *P* = 0.270; Figure 1C) and LRRFS (92.7% vs. 94.7%, *P* = 0.128; Figure 1D) were comparable between these two groups.

After adjusting for various factors, multivariate analysis revealed treatment group (IC-CCRT vs. CCRT-AC) was an independent prognostic factor for 3-year DFS (HR, 1.546; 95% CI, 1.113–2.149; *P* = 0.009) and OS (HR, 1.487; 95% CI, 1.035–2.136; *P* = 0.032). However, it did not have a survival impact on 3-year DMFS (HR, 1.294; 95% CI, 0.847–1.976; *P* = 0.233) and LRRFS (HR, 1.530; 95% CI, 0.869–2.696; *P* = 0.141) (Table 2). Undoubtedly, overall stage (IVA vs. III) was an independent prognostic factor for all endpoints.

**Table 2:** Results of multivariate analysis for the 309 pairs with LA-NPC (except T3N0)

Endpoints	Variables	HR (95% CI)	*P*^a^
DFS	Smoking (Yes vs. no)	1.867 (1.345–2.591)	< 0.001
	Treatment group (IC-CCRT vs. CCRT-AC)	1.546 (1.113–2.149)	0.009
	Overall stage (IVA vs. III)	2.287 (1.610–3.247)	< 0.001
OS	Smoking (Yes vs. no)	2.254 (1.574–3.228)	< 0.001
	Treatment group (IC-CCRT vs. CCRT-AC)	1.487 (1.035–2.136)	0.032
	Overall stage (IVA vs. III)	2.421 (1.641–3.572)	< 0.001
DMFS	Smoking (Yes vs. no)	1.578 (1.028–2.422)	0.037
	Treatment group (IC-CCRT vs. CCRT-AC)	1.294 (0.847–1.976)	0.233
	T category (T3-4 vs. T1-2)	2.266 (1.094–4.693)	0.028
	N category (N2-3 vs. N0-1)	2.096 (1.345–3.266)	0.001
	Overall stage (IVA vs. III)	2.254 (1.415–3.591)	0.001
LRRFS	Treatment group (IC-CCRT vs. CCRT-AC)	1.530 (0.869–2.696)	0.141
	Overall stage (IVA vs. III)	1.647 (0.924–2.934)	0.091

Abbreviations: LA-NPC = locoregionally advanced nasopharyngeal carcinoma; DFS = disease-free survival; OS = overall survival; DMFS = distant metastasis-free survival; LRRFS = locoregional relapse-free survival; HR = hazards ratio; CI = confidence interval; KPS = Karnofsky performance score; IC = induction chemotherapy; CCRT = concurrent chemoradiotherapy; AC = adjuvant chemotherapy.

^a^Multivariate *P*-values were calculated using an adjusted Cox proportional-hazards model with backward elimination and the following parameters: age (> 48 y vs. ≤ 48 y), gender (female vs. male), KPS (≥ 90 vs. ≤ 80), smoking (yes vs. no), drinking (yes vs. no), T category (T3-4 vs. T1-2), N category (N2-3 vs. N0-1), overall stage (IVA vs. III), concurrent chemotherapy regimen (TP vs. PF), treatment group (IC-CCRT vs. CCRT-AC).

### Subgroup analysis

To further establish the survival difference of these treatment modalities in patients at different risk, we therefore conducted stratified analysis according to the tumor stage because it was established as an independent prognostic factor by multivariate analysis.

**Figure 2:**
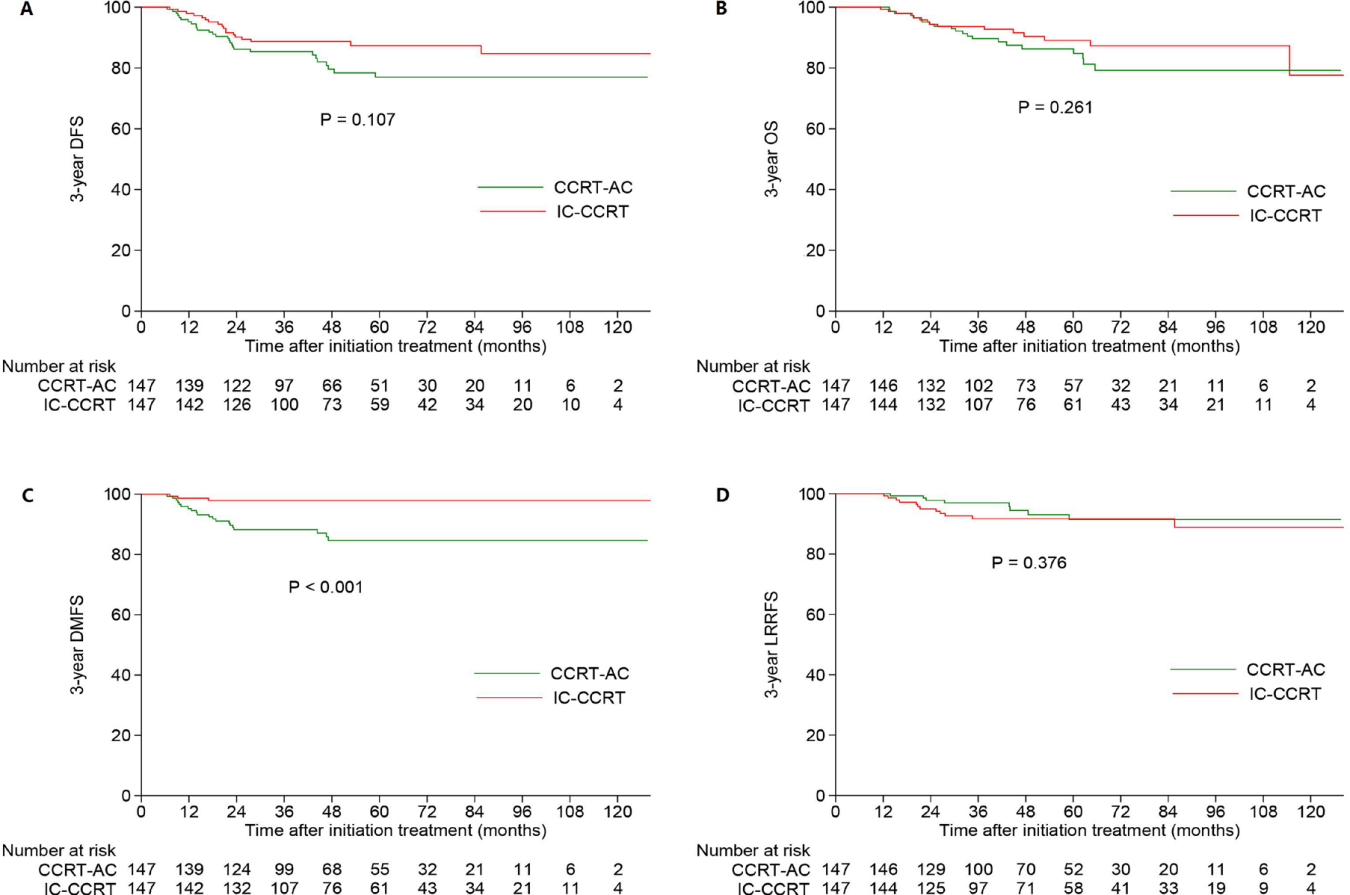
Kaplan-Meier DFS (**A**), OS (**B**), DMFS (**C**) and LRRFS (**D**) curves for patients with stage III receiving IC-CCRT or CCRT-AC. Abbreviations: OS = overall survival; DFS = disease-free survival; DMFS = distant metastasis-free survival; LRRFS = locoregional relapse-free survival; IC = induction chemotherapy; CCRT = concurrent chemoradiotherapy; AC = adjuvant chemotherapy.

In primary cohort, 407 patients had stage III disease and 147 pairs were selected for this analysis. The 3-year DFS, OS, DMFS and LRRFS rates for the IC-CCRT vs. CCRT-AC group were 88.7% vs. 85.4% (*P* = 0.107; Figure 2A), 93.7% vs. 89.7% (*P* = 0.261; Figure 2B), 97.9% vs. 88.3% (*P* < 0.001; Figure 2C) and 91.7% vs. 97.0% (*P* = 0.376; Figure 2D), respectively. When multivariate analysis was performed, IC-CCRT group was found to be superior to CCRT-AC group with regard to DMFS (HR, 0.145; 95% CI, 0.043–0.488; *P* = 0.002), while the other endpoints were comparable between the two groups (Table 3).

**Table 3:** Results of multivariate analysis in different subgroups

Endpoints	Variable	HR (95% CI)	*P*^a^
Stage III cohort			
DFS	N category (N2 vs. N1)	2.030 (1.080–3.815)	0.028
	Treatment group (IC-CCRT vs. CCRT-AC)	0.617 (0.341–1.116)	0.111
OS	KPS (≥ 90 vs. ≤ 80)	0.298 (0.104–0.854)	0.024
	N category (N2 vs. N1)	1.887 (1.045–3.407)	0.035
	Treatment group (IC-CCRT vs. CCRT-AC)	0.704 (0.361–1.373)	0.303
DMFS	N category, N2 vs. N1	2.409 (1.199–4.839)	0.014
	Treatment group (IC-CCRT vs. CCRT-AC)	0.145 (0.043–0.488)	0.002
LRRFS	Treatment group (IC-CCRT vs. CCRT-AC)	1.431 (0.578–3.539)	0.438
Stage IVA cohort			
DFS	Smoking (Yes vs. No)	3.239 (1.820–5.763)	< 0.001
	Treatment group (IC-CCRT vs. CCRT-AC)	2.009 (1.316–3.065)	0.001
	N category (N2-3 vs. N0-1)	1.622 (1.083–2.428)	0.019
OS	Smoking (Yes vs. No)	0.254 (0.09–0.714)	< 0.001
	Treatment group (IC-CCRT vs. CCRT-AC)	1.671 (1.060–2.636)	0.027
	N category (N2-3 vs. N0-1)	1.648 (1.062–2.560)	0.026
DMFS	Smoking (Yes vs. No)	2.210 (1.314–3.718)	0.003
	Treatment group (IC-CCRT vs. CCRT-AC)	1.986 (1.155–3.416)	0.013
	N category (N2-3 vs. N0-1)	2.066 (1.209–3.530)	0.008
LRRFS	Treatment group (IC-CCRT vs. CCRT-AC)	1.445 (0.693–3.013)	0.326

Abbreviations: DFS = disease-free survival; OS = overall survival; DMFS = distant metastasis-free survival; LRRFS = locoregional relapse-free survival; HR = hazards ratio; CI = confidence interval; IC = induction chemotherapy; CCRT = concurrent chemoradiotherapy; AC = adjuvant chemotherapy; KPS = karnofsky performance score.

^a^Multivariate *P*-values were calculated using an adjusted Cox proportional-hazards model with backward elimination and the following parameters: age (> 48 y vs. ≤ 48 y), gender (female vs. male), KPS (≥ 90 vs. ≤ 80), smoking (yes vs. no), drinking (yes vs. no), T category (T3-4 vs. T1-2), N category (N2-3 vs. N0-1), concurrent chemotherapy regimen (TP vs. PF) and treatment group (IC-CCRT vs. CCRT-AC).

**Figure 3:**
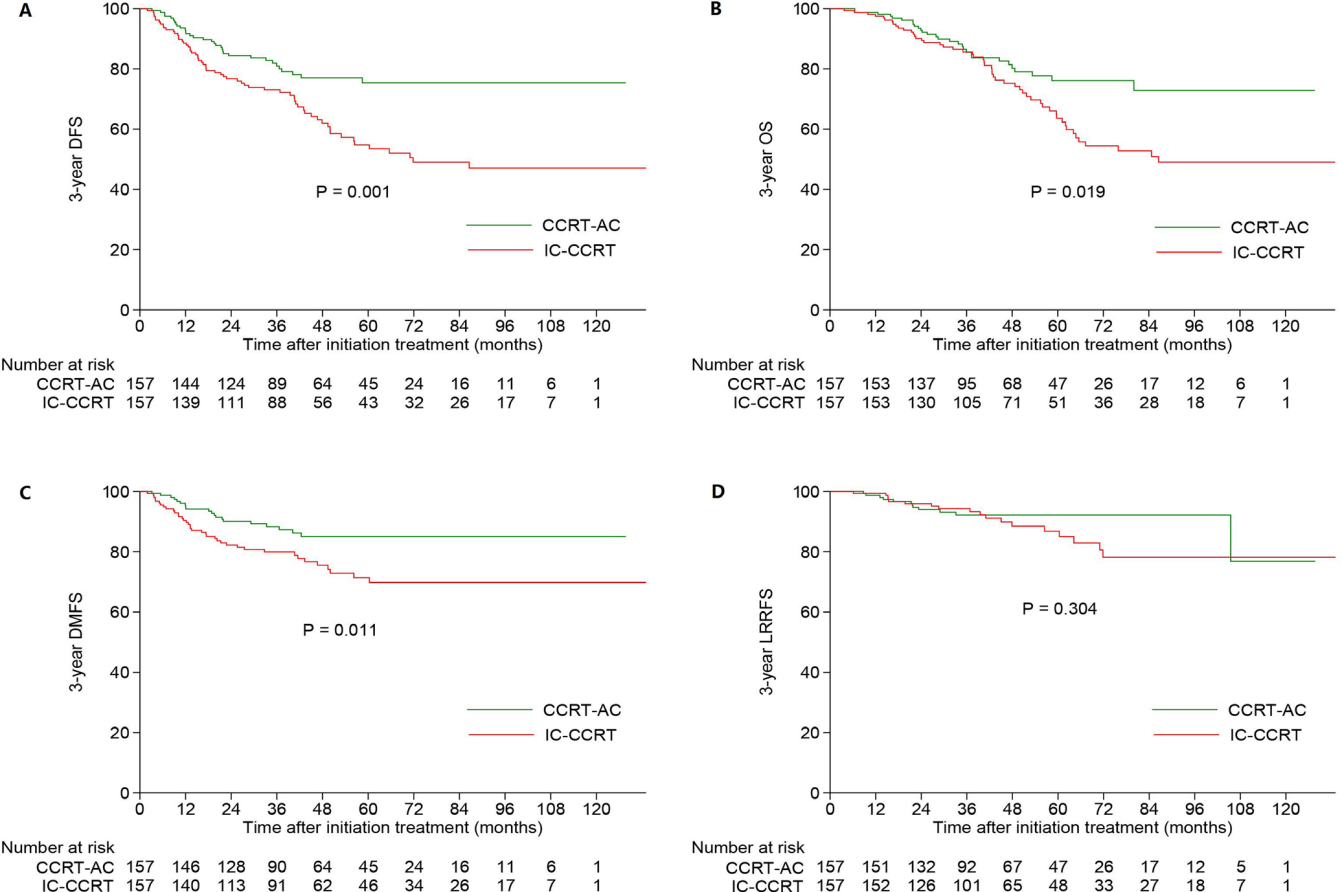
Kaplan-Meier DFS (**A**), OS (**B**), DMFS (**C**) and LRRFS (**D**) curves for patients with stage IVA receiving IC-CCRT or CCRT-AC. Abbreviations: OS = overall survival; DFS = disease-free survival; DMFS = distant metastasis-free survival; LRRFS = locoregional relapse-free survival; IC = induction chemotherapy; CCRT = concurrent chemoradiotherapy; AC = adjuvant chemotherapy.

Among the 427 patients with stage IVA disease, 157 pairs were selected. The 3-year DFS, OS, DMFS, LRRFS rates in the IC-CCRT and CCRT-AC groups were 73.0% and 81.0% (*P* = 0.001; Figure 3A), 85.6% and 85.5% (*P* = 0.019; Figure 3B), 79.9% and 88.4% (*P* = 0.011; Figure 3C), 94.3% and 92.2% (*P* = 0.304; Figure 3D), respectively. Obviously, patients in the CCRT-AC group achieved significantly better DFS, OS and DMFS. And consistent with the results of univariate analysis, treatment group (IC-CCRT vs. CCRT-AC) was established as a survival predictive factor for DFS (HR, 2.009; 95% CI, 1.316–3.065; *P* = 0.001), OS (HR, 1.671; 95% CI, 1.060–2.636; *P* = 0.027) and DMFS (HR, 1.986; 95% CI, 1.155–3.416; *P* = 0.013) but not for LRRFS (HR, 1.445; 95% CI, 0.693–3.013; *P* = 0.326) (Table 3).

## DISCUSSION

Notably, our study to date is the first head-to-head study with the largest cohorts to compare the therapeutic gain achieved by IC-CCRT or CCRT-AC in LA-NPC (except T3N0). The results demonstrated that CCRT-AC chemotherapy sequence was associated with significantly improved DFS and OS compared with IC-CCRT, and this benefit may mainly originate from the cohort of patients with stage IVA disease. With the advantages of employing PSM and multivariate analysis, this study provided the fairest comparisons of matched patients and the outcomes were robust.

CCRT followed by adjuvant fluorouracil with cisplatin has been recommended as the standard treatment strategy for LA-NPC by National Comprehensive Cancer Network (NCCN) guidelines since the publication of Intergroup-0099 trial [5], and many subsequent studies carried out in Asia further established the value of this chemotherapy regimen [9, 10, 12, 27]. However, this regimen brought severe toxicities and the compliance to three cycles in previously reported studies was unsatisfactory (52–63%) [5, 9, 10, 12, 27, 28]. Therefore, a strategy with better efficacy and less toxicities is needed to improve compliance to treatment and systemic control. Consequently, much attention has been paid to induction chemotherapy as it has satisfactory compliance and could shrink tumor volume and eliminate micro-metastasis before radiotherapy. Many trials achieved positive results and made induction chemotherapy a more promising treatment modality [15, 19, 23, 29]. However, little is known about the comparison of these two regimens since no randomized trial has been undertaken except the study consisting of six arms by Lee et al. [20] and two network meta-analysis [25, 26]. In the current study, we presented that CCRT-AC may be a more effective option than IC-CCRT in decreasing death and treatment failure for patients with stage III-IVA (except T3N0) disease. The underlying reason for this difference may be that patients in the IC-CCRT group experience longer wait time before definitive radiotherapy which could do harm to survival [25, 30].

Stratified analysis according to the tumor stage (III or IVA) was performed as it was an independent prognostic factor indicated by multivariate analysis. Intriguingly, absolutely different outcomes were observed in these two subpopulations. In the cohort of stage III, a better DMFS rate was obtained in the IC-CCRT group although the other endpoints were non-significant. However, CCRT-AC could achieved better DFS and OS compared with IC-CCRT in patients with stage IV disease. Two potential factors may contribute to the different results. First, tumor burden and risk of distant metastasis is higher in the stage IVA disease than that in stage III disease. The negative effects of prolonged wait time on survival outcomes may be amplified and more obvious in these patients with higher risk of metastasis. Moreover, compared with the PF regimen used in previous studies [5, 9, 12, 28, 31], the chemotherapy regimen used in our study in the CCRT-AC cohort were TPF and TP which were more effective than the PF regimen. Therefore, it may be likely that TPF or TP works in the adjuvant phase better than in the induction phase. Notably, the DFS rate at the 3-year point was higher in the CCRT-AC group than that in IC-CCRT group but OS rate (85.6% vs. 85.5%) was similar in the stage IVA subpopulation. The reason may be attributed to the effective salvage treatment for patients experienced treatment failure after IC-CCRT. Although OS rate at 3-year point was comparable, a significant difference (*P* = 0.019) was still obtained and that is because more patients experienced treatment failure after 3 years. Therefore, these results may indicate that CCRT-AC treatment still has a strong protective effect after 3 years.

It should be pointed out that the concurrent chemotherapy regimen used in our study is double agents consisting of TP and PF, which originated from the study by Lin et al. [11]. Although, double-agent regimens may had more toxicities than single agent of cisplatin, we only assigned two cycles and most of patients could completed that. Moreover, multivariate analysis reveal the chemotherapy regimen (TP vs. PF) used in concurrent phase was not a prognostic factor for all endpoints. Therefore, the concurrent chemotherapy should have no or very limited impact on the conclusions.

The main strength of this study is the adoption of PSM and multivariate analysis to evaluate the survival difference between patient receiving IC-CCRT and CCRT-AC chemotherapy sequences in LA-NPC; this addressed the potential limitations of divergent confounders, treatment heterogeneity and selection bias associated with retrospective analysis [32]. As with all retrospective studies, the weakness of this study should also be acknowledged. First, the data was collected from a single institution and toxicity data was unavailable because our retrospective study did not collected this. Moreover, prognostic biomarker such as plasma Epstein-Barr virus (EBV) DNA [3, 33–35] was not given consideration because part of the data were collected at a very early time when detection of plasma EBV DNA was unavailable. Future clinical trials regarding plasma EBV DNA should be conducted for better risk stratification when comparing these two treatment modalities.

## MATERIALS AND METHODS

### Patient selection

We retrospectively reviewed data on patients with newly diagnosed, non-metastatic NPC receiving IMRT between July, 2004 and December, 2014 at Nanjing Medical University Affiliated Cancer Hospital of China. Patients meeting the following criteria were recruited for this study: (1) stage III-IVA NPC (except T3N0); (2) World Health Organization (WHO) pathology type II/III; (3) receiving either IC-CCRT or CCRT-AC; (3) age 18–70 years older; (4) without previous malignancy; (5) did not receiving chemotherapy or/and radiotherapy previously. Finally, 834 patients were identified. This study was approved by the Research Ethics Committee of Jiangsu Cancer Hospital. Written informed consent was obtained from all the patients before treatment.

### Staging workup

The routine staging workup before treatment contained a complete history collecting, clinical examinations of the head and neck region and direct fibre-optic nasopharyngoscopy. Radiographic examinations included magnetic resonance imaging (MRI) or contrast-enhanced computed tomography (CT) scans of the skull base and whole neck, chest radiography, whole-body bone scan and abdominal sonography, as well as positron emission tomography (PET)-CT if necessary. All MRI materials and clinical records were reviewed to minimize heterogeneity in restaging, and all patients were restaged according to the 8th edition of the International Union against Cancer/American Joint Committee on Cancer (UICC/AJCC) system [36].

### Radiotherapy

Definitive IMRT was delivered to all the patients with 6 MV X-rays in our center as reported previously [37]. Briefly, gross tumor volume (GTVnx) included the primary tumor and metastatic retropharyngeal lymph nodes. Metastatic cervical lymph nodes were defined as GTVnd. The high-risk region was defined as clinical target volume (CTV1) which included the whole nasopharyngeal cavity, GTVnx, GTVnd with a margin of 5 to 15 mm, and levels II and III cervical lymphatic drainage region. Low risk area was defined as CTV2 which encompassed CTV1 with a margin of 3 to 5 mm, the lower neck, and the supraclavicular lymphatic drainage region. A total prescribed dose of 66–75 Gy/31–35 fractions to the planning target volume (PTV) of GTVnx, 65–75 Gy/32–35 fractions to the PTV of GTVnd, 56–60Gy/30 fractions to the PTV of CTV1 and 50 Gy/30 fractions to the PTV of CTV2, respectively. All patients were irradiated with 1 fraction daily, 5 days per week.

### Chemotherapy

IC or AC were platinum-based chemotherapy regimens including 5-fluorouracil (1000 mg/m^2^ d1-d5) with cisplatin (80 mg/m^2^ in total for 3 days) (PF), docetaxel (75 mg/m^2^ d1) with cisplatin (80 mg/m^2^ in total for 3 days) (TP) or triplet of docetaxel (60 mg/m^2^ d1) plus cisplatin (80 mg/m^2^ in total for 3 days) with 5-fluorouracil (1000 mg/m^2^ d1-d5) (TPF), which were administered every three weeks for 2–3 cycles. Concurrent chemotherapy consisted of two cycles of TP or PF and the dosage was delivered as abovementioned.

### Follow-up

Follow-up duration was measured from first day of pathological diagnosis to last examination or death. Patients were assessed every 3 months during the first 2 years, then every 6 months thereafter (or until death) by clinical examinations, abdominal sonography, MRI of nasopharynx and neck and chest X-ray or CT. PET-CT was also performed if clinical symptoms indicated distant metastasis. End points analysed in our study included 3-year disease-free survival (DFS), overall survival (OS), distant metastasis-free survival (DMFS) and locoregional relapse-free survival (LRRFS).

### Statistical analysis

Propensity score matching (PSM) [32] was computed by logistic regression for each patient using the following covariates: age, gender, karnofsky performance score (KPS), smoking, drinking, T category, N category, overall stage, IC/AC regimen and cycle, and CCRT regimen. Chi-square test or Fisher’s exact test were employed to compare categorical variables and treatment failure patterns between IC-CCRT and CCRT-AC groups. Non-parametric test was used to compare continuous variables. Survival rates were estimated using the Kaplan-Meier method and the difference was compared by log-rank test. The multivariate Cox proportional hazards model was performed to estimate hazard ratios (HRs) and 95% confidence intervals (CIs); age (> 44 y vs. ≤ 44 y), gender (female vs. male), KPS (≥ 90 vs. ≤ 80), smoking (yes vs. no), drinking (yes vs. no), T category (T3-4 vs. T1-2), N category (N2-3 vs. N0-1), overall stage (IVA vs. III), concurrent chemotherapy regimens (TP vs. PF), treatment group (IC-CCRT vs. CCRT-AC). All tests were two-sided; *P* < 0.05 was considered significant. Stata Statistical Package 12 (StataCorp LP, College Station, TX, USA) was used for all analyses.

### Grant support

This work was funded by the National Natural Science Foundation of China (No. 81672989), Jiangsu Clinical medicine Science and Technology Special Fund (BL2014091), and Jiangsu Provincial Commission of Health and Family Planning Youth Research Project (Q201601).

## CONCLUSIONS

Overall, our findings suggest that CCRT-AC treatment modality could achieve better therapeutic outcomes compared with IC-CCRT in the era of IMRT and this benefit may mainly originate from the patients with stage IVA, while IC-CCRT may be considered for stage III disease. Future randomized trials consisting more risk stratification factors are warranted to confirm our results.
